# Effects of Genetic Polymorphisms of Drug Transporter ABCB1 (MDR1) and Cytochrome P450 Enzymes CYP2A6, CYP2B6 on Nicotine Addiction and Smoking Cessation

**DOI:** 10.3389/fgene.2020.571997

**Published:** 2020-11-30

**Authors:** Ahmet Muderrisoglu, Elif Babaoglu, Elif Tugce Korkmaz, Mert C. Ongun, Erdem Karabulut, Alper B. Iskit, Salih Emri, Melih O. Babaoglu

**Affiliations:** ^1^Department of Pharmacology, Faculty of Medicine, Hacettepe University, Ankara, Turkey; ^2^Department of Chest Diseases, Faculty of Medicine, Hacettepe University, Ankara, Turkey; ^3^Department of Biostatistics, Faculty of Medicine, Hacettepe University, Ankara, Turkey

**Keywords:** nicotine, genetic polymorphism, addiction, P-glycoprotein, cytochrome P450 enzymes

## Abstract

**Objectives:**

To determine the effects of genetic polymorphisms of ABCB1 (MDR1), CYP2A6, CYP2B6 on smoking status, and clinical outcomes of smoking cessation therapies in a Turkish population.

**Methods:**

130 smokers and 130 non-smokers were recruited. Individuals who never smoked were described as non-smokers. 130 smokers were treated with nicotine replacement therapy (NRT) (*n* = 40), bupropion (*n* = 47), bupropion + NRT (*n* = 15), and varenicline (*n* = 28). Smokers were checked by phone after 12 weeks of treatment whether they were able to quit smoking or not. Genotyping and phenotyping were performed.

**Results:**

Cessation rates were as follows; 20.0% for NRT, 29.8% for bupropion, 40.0% for bupropion + NRT, 57.1% for varenicline (*p* = 0.013). The frequency of ABCB1 *1236TT-2677TT-3435TT* haplotype was significantly higher in non-smokers as compared to smokers (21.5% vs. 10.8, respectively; *p* = 0.018). Neither smoking status nor smoking cessation rates were associated with genetic variants of CYP2A6 (*p* = 0.652, *p* = 0.328, respectively), or variants of CYP2B6 (*p* = 0.514, *p* = 0.779, respectively).

**Conclusion:**

Genetic variants of the drug transporter ABCB1 and the *1236TT-2677TT-3435TT* haplotype was significantly associated with non-smoking status. Neither ABCB1 nor CYP2A6, CYP2B6 genetic variants were associated with smoking cessation rates at the 12th week of drug treatment.

## Introduction

Tobacco smoking is a major health problem globally. Over one billion people around the world consume tobacco products and an estimated six million people, including children are clinically affected. This number is expected to rise to eight million by the year 2030 (WHO, 2017). Among many chemicals in cigarettes, nicotine is the main ingredient responsible for dependence (Miwa et al., 2011). Nicotine addiction has been classified as the tobacco use disorder according to DSM V criteria (American Psychiatric Association, 2013).

Among current first-line pharmacotherapies in smoking cessation are nicotine replacement therapy (NRT), bupropion, and varenicline. The success of smoking cessation treatments may be altered by some pharmacogenetic factors, including genetic variants of drug metabolizing enzymes or drug transporters (Lerman et al., 2006; Benowitz, 2008; Bergen et al., 2013; Chenoweth and Tyndale, 2017).

ABCB1 (MDR1, P-glycoprotein) is an efflux protein expressed in many tissues including the blood-brain barrier. Its activity is involved in the determination of tissue levels of many endogenous substances and xenobiotics (Choi, 2005; Bruhn and Cascorbi, 2014). Genetic polymorphisms of ABCB1 have been shown to affect clinical responses to drugs (Zhou, 2008; Zoto et al., 2015). Three commonly investigated single nucleotide polymorphisms (SNPs) in the protein coding region *C1236T* (rs1128503), *G2677T/A* (rs2032582), and *C3435T* (rs1045642) have been associated with changes in ABCB1 activity (Bruhn and Cascorbi, 2014; Wolking et al., 2015; Kim et al., 2018). Hoffmeyer et al. (2000) found one of the first evidence on pharmacogenetic influences of ABCB1 activity in humans. They determined that in Caucasians, homozygous mutant carriers for the *C3435T* variant were shown to have a 38% increase in peak steady-state concentrations of digoxin, which is a P-gp substrate, compared to wild-type genotype carriers (Hoffmeyer et al., 2000). This result suggested a reduced activity of ABCB1 related to this variant leading to a decreased intestinal secretion of digoxin. Of these three SNPs, *C1236T* and *C3435T* are silent polymorphisms. While *C1236T* does not affect protein expression or mRNA stability, altered mRNA, and protein expression levels in the duodenum and kidney were reported for *C3435T* polymorphism (Siegsmund et al., 2002; Wang D. et al., 2005). On the other hand, G2677T/A genetic polymorphism is a non-synonymous mutation with substitution of alanine with serine or threonine (Hodges et al., 2011). It has been associated with altered transporter activity (Kim et al., 2001). Investigations on the effects of these three genotypic variants on the pharmacokinetics of P-gp substrates have suggested functionally important yet inconclusive results (Gerloff et al., 2002; Hsin et al., 2020).

Nicotine was reported to alter the expression level or function of ABCB1 (Wang J. S. et al., 2005; Pan et al., 2009; Pal et al., 2011; Takano et al., 2016), which is responsible for the transportation of many endogenous substances, such as opioid peptides, steroid hormones, and endorphin (Oude Elferink and Zadina, 2001). These endogenous or some exogenous ABCB1 substrates may contribute in the formation of substance abuse in the central nervous system (Oude Elferink and Zadina, 2001; Choi, 2005). For example, Levran et al. (2008) showed that ABCB1 function and genetic polymorphisms are involved in heroin abuse. It is conceivable that ABCB1 genetic variants may play a role in nicotine addiction and may be affecting the status of tobacco consumption or outcomes of cessation therapies. Such an association has not been shown in the literature.

Therefore, we aimed to investigate the effects of genetic polymorphisms of ABCB1 on the severity of nicotine dependence and clinical outcomes of smoking cessation therapies in a cohort of Turkish population. Also, genetic variants of metabolizing enzymes CYP2A6 and CYP2B6 which are involved in nicotine metabolism were examined.

## Materials and Methods

### Participants

All procedures performed in studies involving human participants were in accordance with the ethical standards of the Hacettepe University Ethics Committee (Protocol number: GO-16/416-03) and with the 1964 Helsinki declaration and its later amendments or comparable ethical standards. Informed consent was obtained from all individual participants included in the study.

A total of 260 subjects were recruited for the study. Among these subjects, 130 (60 females, 70 males) were smokers who applied to the Smoking Cessation Unit and 130 (73 females, 57 males) were non-smokers as the control group. Individuals who never smoked were described as non-smokers. The age range of subjects was 18–71 years old. Subjects with severe heart, liver, kidney diseases, anxiety disorder according to DSM V, pregnant women, and subjects using a nicotine product other than cigarettes or those having an addiction other than nicotine were excluded from the study. Subjects using drugs that would change CYP2A6 or CYP2B6 activities were also excluded. Smoking status was determined by the Fagerström Test for Nicotine Dependence (FTND) and verified by exhaled CO measurements by using piCO Smokerlyzer (Bedfont Scientific Ltd., Kent, United Kingdom). Subjects with exhaled CO values higher than 4 ppm were evaluated as active smokers. All smokers were treated with the appropriate choice of drug therapies according to the European Network for Smoking and Tobacco Prevention (ENSP) Guidelines for Treating Tobacco Dependence (2016) (ENSP, 2016).

In order to assess the effects of genetic polymorphisms on the severity of nicotine dependence, 260 participants were separated into the following 2 groups as smokers and non-smokers. Smokers were treated with NRT, bupropion, bupropion + NRT, and varenicline, as indicated by the ENSP Guideline. We included both nicotine gum, and nicotine patch users in the NRT group because it was shown that form or dosage of nicotine did not affect smoking cessation rates (Stead et al., 2012).

The success of the smoking cessation was checked by re-calling patients after 12 weeks after the beginning of drug treatment.

### Genotyping

Genomic DNA was extracted using Whole Blood DNA Purification Kit (Thermo Fischer Scientific, Waltham, MA, United States) from venous blood. Genotyping was performed by a Hot Start PCR-RFLP method for the *ABCB1* 2677G > T/A-3435C > T, *CYP2A6*^∗^1, ^∗^4, and *CYP2B6* rs2279343 genetic polymorphisms as described previously (Cascorbi et al., 2001; Nakajima et al., 2006; Tomaz et al., 2015). An allele-specific PCR method was used to genotype *CYP2A6*^∗^9 polymorphism (Nakajima et al., 2006). For the *ABCB1* 1236C > T genetic polymorphism, new PCR primers were designed. A total volume of 25 μl containing 200 μM of each dATP, dCTP, dGTP, dTTP, 2.5 mM MgCl_2_, BSA, and 12.5 pmol of each primer, 1 unit of Taq DNA Polymerase, and 100 ng of genomic DNA used as PCR mixture (Solis BioDyne, Tartu, Estonia). Hot start PCR conditions were; 95°C for 15 min following 30 cycles of 95°C for 20 s, 60°C for 30 s, 72°C for 1 min, and 72°C for 10 min. PCR cycles were performed by a thermal cycler (Bio-Rad T100 Thermal Cycler, Bio-Rad Laboratories, Taipei, Taiwan). PCR products were restricted by restriction enzymes (New England Biolabs, Ipswich, MA, United States). Restriction fragments were separated by 3% agarose gel electrophoresis and visualized under UV light (Kodak, Rochester, NY, United States). Table 1 shows the PCR primers, restriction enzymes, and restriction conditions (Tufan et al., 2007; Zoto et al., 2015; Karaca et al., 2017).

**TABLE 1:** Details of genotyping methods.

**Genetic polymorphism**	**PCR primers**	**PCR product**	**Restriction enzymes**	**Restriction conditions**	**Restriction fragments**
*ABCB1* 1236C > T	5′-TGG ACT GTT GTG CTC TTC CC-3′	455 bp	*Hae*III	37°C for 1 h	377 + 43 + 35 bp for C allele
	5′-TGT CAC TTT ATC CAG CTC TCC A-3′				412 + 43 bp for T allele
*ABCB1* 2677G > T	5′-TGC AGG CTA TAG GTT CCA GG-3′	224 bp	*Ban*I	65°C for 1 h	198 + 26 bp for G allele
	5′-TTT AGT TTG ACT CAC CTT CCC G-3′				224 bp for T allele
*ABCB1* 2677G > A	5′-TGC AGG CTA TAG GTT CCA GG-3′	220 bp	*Bsr*I	37°C for 1 h	220 bp for T allele
	5′-GTT TGA CTC ACC TTC CCA GG-3′				206 + 14 bp for A allele
*ABCB1* 3435C > T	5′-TGT TTT CAG CTG CTT GAT GG-3′	197 bp	*Mbo*I	37°C for 1 h	158 + 39 bp for C allele
	5′-AAG GCA TGT ATG TTG GCC TC-3′				197 bp for T allele
CYP2A6*1A/*1B/*4	5′-CAC CGA AGT GTA CCC TAT GCT G-3′	1,338 bp	*Bsu*36I + *Bst*UI	37°C for 1 h and 60°C for 1 h	800 + 434 + 104 bp for the *1A allele
	5′-AAA ATG GGC ATG AAC GCC C-3′				800 + 285 + 149 + 104 bp for the *1B allele
					759 + 285 + 149 + 104 + 41 bp for the *4 allele
*CYP2A6* *9	5′-TCC CTC TTT TTC AGG CAG GCA GTA G-3′ (F-WT*)	385 bp			*9
	5′-TCC CTC TTT TTC AGG CAG GCA GTA T-3′ (F-M**)				
	5′-TCC TGT CTT TCT GAT GCT GA-3 (R)				
*CYP2B6* rs2279343	5′-GAC AGA AGG ATG AGG GAG GAA-3′	640 bp	*Sty*I	37°C for 1 h	297 + 171 + 116 + 56 bp for the A allele
	5′-CTC CCT CTG TCT TTC ATT CTG T-3′				468 + 116 + 56 bp for the G allele

*F-WT, forward primer for the *1 wild type PCR product; **F-M, forward primer for the *9 polymorphic PCR product; R, reverse primer.

### Measuring Plasma Cotinine and *Trans-*3′-Hydroxycotinine Levels

Plasma cotinine and *Trans-*3′-hydroxycotinine (3-HC) levels were measured by high performance liquid chromatography (HPLC) as described by a previous study (Malaiyandi et al., 2006). Agilent Systems 1200 Series (Agilent Technologies, Santa Clara, CA, United States) liquid chromatography system was used. Chemicals were acquired from Sigma-Aldrich Chemie GmbH (Taufkirchen, Germany). Samples were measured at the 260 nm wavelength. 0–100–250–500–1,000 ng/ml cotinine and 3-HC solutions were used for method validation. The correlation of the calibration curve fit was calculated larger than 0.9999 for both metabolites. The detection limits of the HPLC-UV assay for cotinine and 3-HC were found to be 50 ng/ml and 25 ng/ml, respectively. Inter- and intraday accuracies were calculated to be between 97.9–102.4% for 3-HC, and 93.4–102.2% for cotinine (*n* = 6). Autosampler stability was at least 24 h at 4°C.

Nicotine metabolite ratio (NMR = plasma 3-HC/cotinine ratios) was used to determine CYP2A6 enzyme activity.

### Statistical Analyses

Allele, genotype, and haplotype frequencies among groups were compared by using Chi-Square and Fischer’s exact tests. Comparisons of the distributions of the variables were performed by using Kruskal–Wallis and Mann–Whitney *U* tests, where applicable. *p* values under 0.05 were accepted as statistically significant *post hoc* analyses were performed and adjusted *p* values by using Dunn’s test. Statistical analyses were performed by using GraphPad Prism version 6.01 for Windows (GraphPad Software, La Jolla, CA, United States).

For estimation of *ABCB1* haplotype frequencies and their effects on smoking status, the internet resource SNPStats^1^ was used as an *in silico* statistical tool.

The datasets during and/or analyzed during the current study are available from the corresponding author on reasonable request.

## Results

### Demographics and Treatment Results

The demographics and characteristics of subject groups are given in Table 2. There were no statistically significant differences between smokers and non-smokers among genders and ages. Distribution of the FTND results among the patients treated for nicotine dependence were shown in Supplementary Figure 1. Cigarettes per day and exhaled CO levels (mean ± standard error of means) were 23.7 ± 0.9 and 14.2 ± 0.8 ppm for smokers, respectively. The distribution of all genetic variants examined were in agreement with the Hardy–Weinberg equilibrium (*p* > 0.05).

**TABLE 2:** Characteristics and demographics of participants.

	**Smokers n, (%) (Total *n* = 130)**	**Non-smokers n, (%) (Total *n* = 130)**	***p* values**
**Gender**			
Male	70, (53.8)	57, (43.8)	0.11
Female	60, (46.2)	73, (56.2)	
Age	39.2 ± 1.1	41.6 ± 1.1	0.12

*Age data are shown as mean ± SEM.*

Among 130 smokers, 40 were treated with NRT, 47 with bupropion, 15 with bupropion + NRT, and 28 with varenicline. 12 weeks after the beginning of the treatment, a total of 44 (33.8%) smokers were able to quit smoking. Smoking cessation rates for subgroups are summarized in Table 3.

**TABLE 3:** Smoking cessation rates among treatment modalities.

	**Total: *n*/subtotal *n*, (%)**
NRT	8/40, (20)
Bupropion	14/47, (29.8)
Bupropion + NRT	6/15, (40)
Varenicline	16/28, (57.1)
Total	44/130, (33.8)
χ^2^, df, *p*	10.81, 3, 0.013

### Effect of ABCB1 Genetic Polymorphisms

The frequencies of the variant alleles are shown in Table 4. The distributions of alleles for the *ABCB1* C1236T, G2677T/A, and C3435CT polymorphisms were significantly different between smokers and non-smokers (*p* = 0.005, 0.001, and 0.035 respectively).

**TABLE 4:** Allele frequencies for *ABCB1* genetic polymorphisms.

**Genetic polymorphisms**	**Allele**	**Smokers: *n*, (%) (Total *n* = 130)**	**Non-smokers: *n*, (%) (Total *n* = 130)**	**χ^2^, df, *p***
*ABCB1* 1236C > T	C	150, (57.7)	118, (45.4)	7.88, 1, 0.005
	T	110, (42.3)	142, (54.6)	
*ABCB1* 2677G > T/A	G	156, (60)	119, (45.8)	10.6, 1, 0.001
	T/A	104, (40)	141, (54.2)	
*ABCB1* 3435C > T	C	148, (56.9)	124, (47.7)	4.44, 1, 0.035
	T	112, (43.1)	136, (52.3)	

**FIGURE 1:**
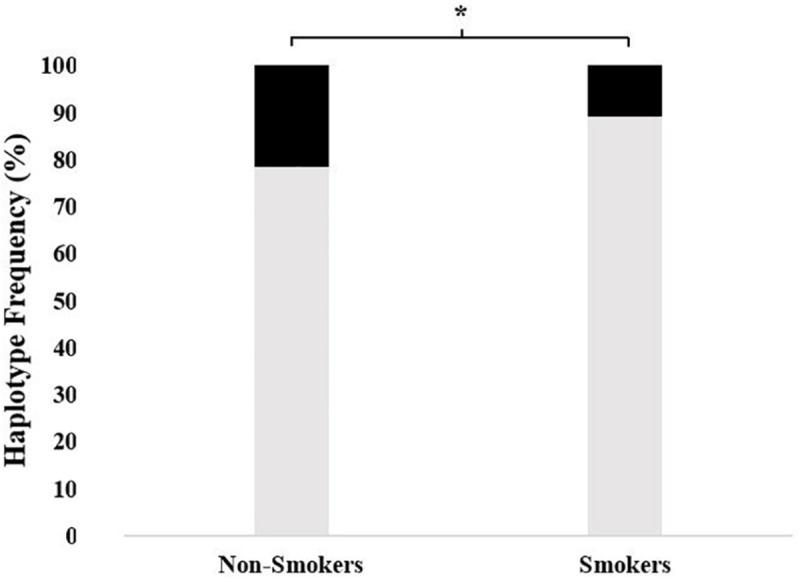
The frequency of 1236TT-2677TT-3435TT haplotype in non-smokers as compared to smokers. The frequencies were 21.5% (95% CI = 14.5–30.3) vs. 10.8% (5.4–16.7), respectively; χ^2^ = 5.57, df = 1, and *p = 0.018) (■: 1236TT-2677TT-3435TT, 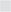: Other haplotypes).

As shown in Figure 1, the frequency of all-variant haplotype for ABCB1 (*1236TT-2677TT-3435TT*) was significantly higher in non-smokers as compared to that in smoker subjects (21.5% vs. 10.8, respectively; χ^2^ = 5.57, df = 1, *p* = 0.018).

Estimated haplotype distributions and their association with smoking status obtained from *in silico* analysis by using the web tool SNPStats are provided in Table 5. In accordance with the findings of Figure 1, the subjects who were carriers of the all-variant haplotype (TTT) of ABCB1 were significantly higher in the non-smoker group and this haplotype was significantly associated with smoking status (Table 5, odds ratio = 0.59, confidence interval 95% CI: 0.40–0.88, and *p* = 0.0099).

**TABLE 5:** Estimated haplotype frequencies and their possible association with smoking status.

**C1236T**	**G2677T**	**C3435T**	**Frequency in smokers**	**Frequency in non-smokers**	**Odds ratio (95% CI)**	***p* value**
C	G	C	0.4712	0.3968	1	-
T	T	T	0.3296	0.469	0.59 (0.40–0.88)	0.0099
C	G	T	0.0857	0.0454	1.53 (0.70–3.35)	0.29
T	T	C	0.0503	0.0616	0.70 (0.31–1.59)	0.4
T	G	C	0.0431	0.0156	2.44 (0.74–8.10)	0.15
C	T	T	0.0155	0.0088	1.49 (0.25–8.90)	0.66
C	T	C	0.0046	0.003	1.25 (0.04–34.85)	0.9
T	G	T	0	0	–	–

*Overall haplotype association *p*-value = 0.02.*

There was no association between genetic polymorphisms of ABCB1 and smoking cessation rates (*p* > 0.05). The haplotypes of *ABCB1* and the corresponding smoking cessation rates are presented in Supplementary Table 1.

### Effect of CYP2A6 or CYP2B6 Genetic Polymorphisms

Allele frequencies for the *CYP2A6^∗^1, ^∗^4*, and *^∗^9* variants were 81.9, 3.0, and 0.5% in all participants, respectively. The variant allele for the *CYP2B6* rs2279343 polymorphism was determined to be 32.9%. The subjects were grouped according to assumed enzyme activities for CYP2A6. *CYP2A6^∗^1/^∗^1* genotype carriers were classified as fast metabolizers, while *^∗^1/^∗^9* genotype carriers as intermediate metabolizers, and *^∗^1/^∗^4*, *^∗^4/^∗^4*, *^∗^4/^∗^9*, and *^∗^9/^∗^9* carriers as slow metabolizers. There was no statistically significant difference between smokers and non-smokers among groups of CYP2A6 activity (*p* > 0.05). There was also no association between *CYP2B6 rs2279343* polymorphism and smoking status (*p* > 0.05).

Our findings yielded no association between smoking cessation rates and *CYP2A6^∗^1/^∗^4/^∗^9* or *CYP2B6* rs2279343 genetic polymorphisms (*p* > 0.05). Data are supplied in Supplementary Table 2.

### Plasma Cotinine, *Trans-*3′-Hydroxycotinine Levels and Nicotine Metabolite Ratios

**FIGURE 2:**
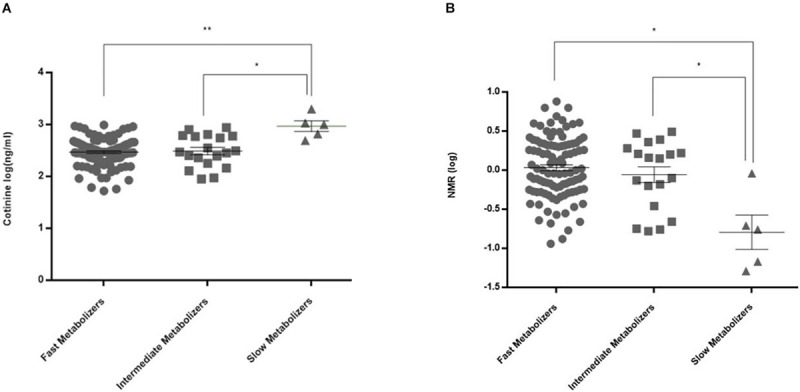
Plasma cotinine levels and NMR values in CYP2A6 activity subgroups. **(A)** Cotinine levels were 348 ± 20 (95% CI = 308–387) ng/ml in fast metabolizers (*n* = 104), 383 ± 56 (266–501) ng/ml in intermediate metabolizers (*n* = 19), and 1,046 ± 216 (490–1,601) ng/ml in slow metabolizers (*n* = 5) (**adjusted *p* = 0.0024, *adjusted *p* = 0.0105). **(B)** NMRs in CYP2A6 activity subgroups were 1.57 ± 0.14 (95% CI = 1.29–1.85) in fast metabolizers (*n* = 102), 1.28 ± 0.22 (0.82–1.74) in intermediate metabolizers (*n* = 19), and 0.34 ± 0.20 (–0.28–0.96) in slow metabolizers (*n* = 4) (*p = 0.04).

For *CYP2A6* genetic polymorphisms, there was a significant difference among the fast metabolizers, intermediate metabolizers, and slow metabolizers in measured cotinine levels and NMR (Figures 2A,B; *p* = 0.004 and 0.04, respectively). In addition, the polymorphic GG genotype for the *CYP2B6* rs2279343 polymorphism was associated with a higher cotinine level (*p* = 0.033, data not shown). Cotinine levels were 373 ng/ml (95% CI = 323–423) in AA + AG genotype carriers, 451 ng/ml (95% CI = 336–565) in GG genotype carriers.

We found no statistically significant differences in cotinine, 3-HC levels, and NMR between quitters and non-quitters (*p* > 0.05). We also found no statistically significant differences in cotinine, 3-HC levels, and NMR between subjects with the all-variant haplotype *1236TT-2677TT-3435TT* as compared with other haplotypes. Data are presented in Supplementary Table 3.

## Discussion

Our results showed that the genetic polymorphisms of the drug transporter ABCB1 (MDR1) were significantly associated with non-smoking status in a cohort of Turkish subjects. We also found that an all-variant haplotype *1236TT-2677TT-3435TT* was about two times more common in non-smokers as compared to smokers (Figure 1). To our knowledge, this is the first study to report an association between ABCB1 genetic polymorphisms and smoking status.

ABCB1 *C1236T* and *C3435T* polymorphisms are silent mutations, albeit with functional consequences. For example, *C1236T* has been associated with methadone doses required for effective treatment of heroin dependence (Levran et al., 2008). Decreased protein and mRNA expression levels of P-gp in the duodenum and kidney were found in carriers of the variant allele for the *C3435T* polymorphism (Siegsmund et al., 2002; Wang D. et al., 2005). *G2677T/A* polymorphism was associated with substitution of alanine with serine or threonine and altered transporter activity (Kim et al., 2001). An interesting and recent population pharmacokinetics study in humans by Hsin et al. (2020) investigated *in vivo* functional effects of common SNP combinations and haplotypes of the ABCB1 gene by measuring the apparent bioavailability of digoxin, which is an ABCB1 substrate. They reported a 35% higher bioavailability of this substrate in individuals carrying the 1236TT-2677TT-3435TT haplotype as compared to those carrying the wild type haplotype (Hsin et al., 2020). They suggested a reduced ABCB1 transporter activity in the gastrointestinal system associated with increased bioavailability of digoxin. Their results may conform with our findings since it provides biological plausibility for our finding of about two-times higher frequency of the all-variant TT-TT-TT haplotype in non-smokers since ABCB1 has been implicated in the transportation of endogenous compounds such as opioid peptides, steroids, glutamate, and endorphin, which act as neuronal modulators in the central nervous system, and which may play roles in mechanisms of substance dependence (Oude Elferink and Zadina, 2001; Choi, 2005; Zhou, 2008). Indeed, the effects of ABCB1 polymorphisms on substance abuse has been reported previously. For example, genetic polymorphisms of ABCB1 affected the dosage of methadone in the treatment of opioid or heroin addiction (Levran et al., 2008; Yuferov et al., 2010). Also, nicotine was shown to alter ABCB1 expression (Pal et al., 2011; Takano et al., 2016) and 4-methylnitrosamino-1–3-pyridyl-1-butanone, which is an ingredient in tobacco smoke, to induce *ABCB1* mRNA expression (Nieh et al., 2015). Therefore, it may be conceivable that ABCB1 genetic polymorphisms may interact with mechanisms related to substance dependence, including nicotine addiction. However, our results do not allow us to draw a direct conclusion since molecular mechanisms related to biological mechanisms have not been investigated.

Our findings also showed that there was no association found between *ABCB1* genetic variants and the success of drug treatments for smoking cessation. The finding of a higher level of cotinine in the *2677TT* sub-group may be a coincidental finding since existing literature do not largely support the view that *ABCB1* variants largely affect plasma concentrations of drugs (Bruhn and Cascorbi, 2014).

Earlier studies reported that CYP2A6 enzyme activity affected nicotine dependence and cigarette consumption (Lerman et al., 2006; Benowitz, 2008; Chenoweth and Tyndale, 2017). Its activity was also found to alter cessation rates during NRT and drugless interventions (Lerman et al., 2006; Schnoll et al., 2009; Chenoweth and Tyndale, 2017; Tanner and Tyndale, 2017). Twin studies indicated that 60–80% of the variation in CYP2A6 enzyme activity can be linked to genetic factors (Swan et al., 2009; Loukola et al., 2015). Asian populations tend to have a higher frequency of polymorphic alleles which cause loss of function (Benowitz et al., 2002; Park et al., 2016). Similarly, African-Americans have lower enzyme activity as compared to Caucasians (Nakajima et al., 2006). We found the frequencies of *^∗^4* and *^∗^9* alleles, which result in activity loss, were lower in our patient population as compared to Asian populations (0.5 and 3%, respectively). Previous studies found that smokers who have slower enzyme activity benefited more from NRT when compared to smokers with high enzyme activity (Lerman et al., 2006; Schnoll et al., 2009). Thus, it was suggested that individuals with high enzyme activity should be treated with varenicline (Lerman et al., 2015). Unlike previous studies (Chenoweth and Tyndale, 2017; Tanner and Tyndale, 2017), our study found no association between high or low enzyme activity groups for CYP2A6 activity and the outcome of cessation. This finding may be due to the population difference in these studies. Our study was limited for not being able to examine *^∗^2* and *^∗^12* polymorphisms of CYP2A6 (Benowitz et al., 2006). Our finding of an association between plasma cotinine levels, or NMRs and assumed CYP2A6 enzyme activities are in agreement with previous findings (Tanner and Tyndale, 2017; Figures 2A,B).

CYP2B6 is the main metabolizing enzyme for bupropion and has a minor role in nicotine metabolism (Thorn et al., 2010). The expression of CYP2B6 in the brain might be related to a role in nicotine dependence (Garcia et al., 2015). It was reported that there was a reduced enzyme activity in carriers of the single nucleotide variant rs2279343 of *CYP2B6* (Al Koudsi and Tyndale, 2010). A study conducted on African-American smokers demonstrated that *CYP2B6^∗^6* haplotype, which included rs2279343 and rs3745274 variants was associated with poorer clinical outcomes with bupropion treatment (Zhu et al., 2012). The same study suggested that higher bupropion doses should be preferred in carriers for the *CYP2B6^∗^6* (516G > T and 785A > G) haplotype. In addition, the same haplotype was associated with increased risk of relapse after cessation in Caucasians (Lee et al., 2007). We found the frequency of variant allele *CYP2B6* rs2279343 was 32.9% in our study population, supporting a previous study conducted on an another Turkish population (Yuce-Artun et al., 2014). Our study showed no association of *CYP2B6* rs2279343 polymorphism with smoking status or cessation rates. Our study was limited for not being able to examine *^∗^9* polymorphism of *CYP2B6*. It is another limitation of our study that it was conducted on a relatively small sample size of subjects. Therefore, our findings need to be confirmed by other studies in various populations.

In conclusion, we found for the first time in the literature that genetic variants of the drug transporter ABCB1 (MDR1) and an all-variant haplotype (*1236TT-2677TT-3435TT*) were significantly associated with non-smoking status. This haplotype and the related allelic variants may serve as a biomarker for protection against nicotine addiction if confirmed by other studies in different populations.

## Data Availability Statement

The original contributions presented in the study are publicly available. This data can be found here: Mendeley Data; doi: 10.17632/f8xhczy3f9.1.

## Ethics Statement

The studies involving human participants were reviewed and approved by Hacettepe University Ethics Committee (Protocol number: GO-16/416-03). The patients/participants provided their written informed consent to participate in this study.

## Author Contributions

AM, EB, and MB contributed to the conception and design of the study. EB, AM, SE, and ETK recruited the subjects. AM, EB, MB, MO, and ETK contributed to the acquisition of the data. AM, EB, MB, SE, AI, and EK contributed to the interpretation of data. All authors contributed to the interpretation of the results and manuscript preparation.

## Conflict of Interest

The authors declare that the research was conducted in the absence of any commercial or financial relationships that could be construed as a potential conflict of interest.

## Footnotes

1 https://www.snpstats.net/start.htm
