# Supplementary material for: Effects of Genetic Polymorphisms of Drug Transporter ABCB1 (MDR1) and Cytochrome P450 Enzymes CYP2A6, CYP2B6 on Nicotine Addiction and Smoking Cessation
Source: Front Genet. 2020 Nov 30;11:571997. doi: 10.3389/fgene.2020.571997 (PMC7734344; doi:10.3389/fgene.2020.571997)
Supplement: Supplementary file 1 [file Data_Sheet_1.docx]

**SUPPLEMENTARY MATERIAL**

**Figure S1.** Distribution of the FTND results among the patients treated for nicotine dependence

**Table S1.** Smoking cessation rates for the *ABCB1* haplotypes

|  | **TT-TT-TT: n, (%)** | **Other Haplotypes: n, (%)** | **χ2; df; p** |
| --- | --- | --- | --- |
| **Quitters** | 3, (6.8) | 41, (93.2) | 1.08, 1, 0.38 |
| **Non-Quitters** | 11, (12.8) | 75, (87.2) |  |

**Table S2.** Drug treatment results in CYP2A6 groups according to the assumed activity levels

|  | **Fast Metabolizers: n; (%)** | **Intermediate Metabolizers: n; (%)** | **Slow Metabolizers: n; (%)** | **χ2; df; p** |
| --- | --- | --- | --- | --- |
| **Quitters** | 39; (88.6) | 4; (9.1) | 1; (2.3) | 3.24; 2; 0.198 |
| **Non- Quitters** | 57; (75) | 15; (19.7) | 4; (5.3) |  |

**Table S3.** Comparison of trans-3'-hydroxycotinine (THC)-cotinine (C) levels and nicotine metabolite ratios (NMR) between *ABCB1* TT-TT-TT haplotype carriers and carriers of other haplotypes

|  | **TT-TT-TT Haplotype Carriers** | **Other Haplotype Carriers** | | **p Value** |
| --- | --- | --- | --- | --- |
| **THC (ng/ml)** | 444±89.4 (250-637) | 505±60.9 (95% CI: 384-627) | 0.75 | |
| **C (ng/ml)** | 482±82 (305-659) | 391±25.4 (340-441) | 0.5 | |
| **NMR (ng/ml)** | 1.05±0.21 (0.59-1.5) | 1.47±0.16 (1.16-1.78) | 0.35 | |

Results are presented as mean±standart error of means (95% Confidence Internal). p values were calculated after measurement results were converted to logarithmic results.
